# A Lightweight Double-Stage Scheme to Identify Malicious DNS over HTTPS Traffic Using a Hybrid Learning Approach

**DOI:** 10.3390/s23073489

**Published:** 2023-03-27

**Authors:** Qasem Abu Al-Haija, Manar Alohaly, Ammar Odeh

**Affiliations:** 1 Department of Cybersecurity, Princess Sumaya University for Technology (PSUT), Amman 11941, Jordan; 2 Department of Information Systems, College of Computer and Information Sciences, Princess Nourah bint Abdulrahman University, P.O. Box 84428, Riyadh 11671, Saudi Arabia; 3 Department of Computer Science, Princess Sumaya University for Technology (PSUT), Amman 11941, Jordan

**Keywords:** cybersecurity, Domain Name System (DNS), DNS over HTTPS (DoH), artificial intelligence, machine learning

## Abstract

The Domain Name System (DNS) protocol essentially translates domain names to IP addresses, enabling browsers to load and utilize Internet resources. Despite its major role, DNS is vulnerable to various security loopholes that attackers have continually abused. Therefore, delivering secure DNS traffic has become challenging since attackers use advanced and fast malicious information-stealing approaches. To overcome DNS vulnerabilities, the DNS over HTTPS (DoH) protocol was introduced to improve the security of the DNS protocol by encrypting the DNS traffic and communicating it over a covert network channel. This paper proposes a lightweight, double-stage scheme to identify malicious DoH traffic using a hybrid learning approach. The system comprises two layers. At the first layer, the traffic is examined using random fine trees (RF) and identified as DoH traffic or non-DoH traffic. At the second layer, the DoH traffic is further investigated using Adaboost trees (ADT) and identified as benign DoH or malicious DoH. Specifically, the proposed system is lightweight since it works with the least number of features (using only six out of thirty-three features) selected using principal component analysis (PCA) and minimizes the number of samples produced using a random under-sampling (RUS) approach. The experiential evaluation reported a high-performance system with a predictive accuracy of 99.4% and 100% and a predictive overhead of 0.83 µs and 2.27 µs for layer one and layer two, respectively. Hence, the reported results are superior and surpass existing models, given that our proposed model uses only 18% of the feature set and 17% of the sample set, distributed in balanced classes.

## 1. Introduction

**Figure 1:**
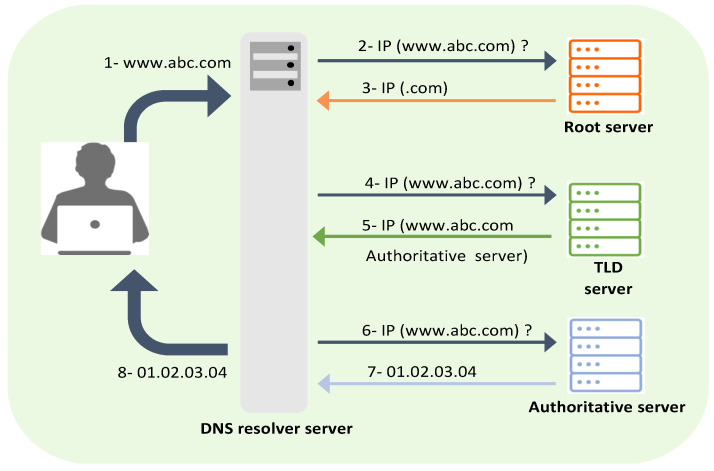
DNS Name Resolution.

Every host on the Internet has a unique IP address that allows users to connect and communicate with it. In the early days of the Internet, users could only access a web server using the server’s IP address. For instance, to visit the Google website, a user must type the server’s IP address, 142.250.178.142, instead of www.google.com. Later, in the 1980s, the number of Internet hosts grew to hundreds of thousands. As a result, it became impractical to memorize and maintain the IP address of every single host on this network [1]. Paul Mockapetris solved this problem by introducing the Domain Name System (DNS). This name resolution system maps a hostname to its IP address [2]. As initially designed, DNS has a hierarchical tree structure consisting of three layers: the root layer, the top-level domain (TLD) layer, and the authoritative layer [2]. The name-to-IP mapping process begins when Internet clients such as web browsers initiate a DNS request and send it to the resolver [2]. The resolver passes the request through different servers to look up the corresponding IP address and send it back to the client, as shown in Figure 1.

By design, DNS traffic is unencrypted. Such plaintext communication allows attackers to launch attacks on the transmitted DNS packets [3]. According to the IDC DNS threat survey report, 88% of the organizations have experienced a DNS-based attack during 2022, with an average of seven attacks per organization. The report also revealed that DNS phishing, DNS hijacking/spoofing, DNS-based malware, and DoS/DDoS attacks were the most common DNS-based attacks. As a result, organizations lost an average of $942,000 per attack [4].

Encrypting DNS traffic prevents malicious attackers from intercepting the communication between the end user and the DNS resolver. To improve DNS security, researchers have proposed two protocols: DNS over TLS and DNS over HTTPS, DoT, and DoH, respectively [5,6]. DNS over TLS (DoT) is a security protocol that embeds the DNS request and response into the standard Transport Layer Security packet (TLS). Using DoT, a web client initiates a TLS session with the resolver, verifies its public key certificates, and calculates the secret key. Once the session is established, the encrypted DNS traffic is exchanged between both parties over a dedicated port (853) [5].

Similarly, DNS over HTTPS (DoH) encrypts DNS traffic to preserve the confidentiality and integrity of DNS communications. Unlike DoT, DoH, which is more recent, does not pass DNS data with TLS traffic but through HTTPS messages. These HTTPS messages are sent over port 443 like typical HTTPS traffic [6].

With the DoT protocol, the dedicated port supports traditional port-based filtering. It allows network administrators to monitor and block DNS traffic to defend against network adversaries while maintaining the confidentiality of DNS communication. However, it has the disadvantage of exposing the dedicated port to attackers and malicious actors. Hence, an attacker can flood the dedicated port (853) with traffic to shut down the DoT communications. On the other hand, adopting the DoH protocol, which embeds DNS traffic within regular HTTPS queries, makes DNS communication less visible to traditional port-based filtering tools. The lack of visibility over the network indicates that attacks can go undetected. Hence, adversaries may exploit the DoH protocol to create covert channels with external command and control servers, perform data exfiltration, etc. [7]. Therefore, we set this work to analyze DoH communication and identify malicious traffic. We summarize the contributions of this work as follows:

- We use a hybrid learning approach to design and implement a lightweight, double-stage anomaly-based IDS to identify malicious DoH traffic.
- We reduce the dimensionality of the feature set using PCA and balance the dataset using RUS to attain a high-performance model using only 18% of the feature set and 17% of the sample set distributed in balanced classes.
- We report on the performance of three supervised learning methods (Adaboost trees, random fine trees, and support vector machines) for DoH IDSs using the CIRA-CIC-DoHBrw-2020 dataset.
- We thoroughly evaluate the developed DoH IDS models using typical evaluation metrics, including accuracy, sensitivity, specificity, F-score, prediction time, classification error, confusion matrix, sensitivity matrix, and precision matrix.
- We contrast our best findings with state-of-the-art DoH IDS models and demonstrate that our hybrid learning-based DoH IDS is better than any former.

The remainder of this paper is organized as follows: Section 2 provides background information, and Section 3 reviews, compares, and summarizes the related work. We then introduce the proposed two-stage DoH malicious traffic detection framework in Section 4. Next, we report and discuss the experiential results in Section 5. Finally, Section 6 concludes our study with recommendations for future work.

## 2. Background

DNS resolvers convert human-readable domain names into machine-readable IP addresses, serving as the Internet’s equivalent of a phone book [8]. DNS requests and replies are typically sent in plaintext (through UDP), implying that anyone able to monitor transmissions, including network administrators and ISPs. Even if a website employs HTTPS, the DNS query itself is unencrypted [9,10]. Because DNS requests are not encrypted, it is easier for attackers to follow users’ online activities. This lack of privacy significantly affects security and, in some situations, human rights [11]. Unencrypted DNS queries can be compared to postcards sent through the mail since anyone who handles the mail might have text printed on the back. As a result, it is not advisable to transmit a postcard containing sensitive or private information [12].

**Figure 2:**
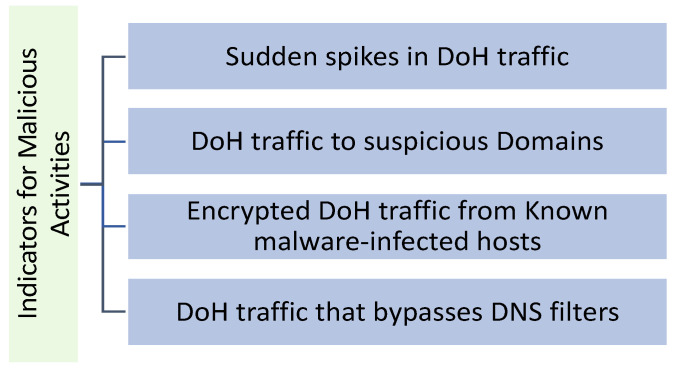
Indicators of malicious activities.

Malicious DNS over HTTPS (DoH) can be challenging to identify because it is designed to look like regular DoH traffic [13]. Figure 2 shows a few indicators that can suggest malicious activity.

According to the figure, the indicators of malicious activities include:

- Sudden spikes in DoH traffic: a sudden increase in network traffic could indicate malicious actors are using DoH to bypass DNS filters [14].
- DoH traffic to suspicious domains: if a lot of DoH traffic goes to domains known to be associated with malware, phishing, or other types of malicious activity, it could be a sign that malicious actors are using DoH to access those domains [15].
- Encrypted DoH traffic from known malware-infected hosts: if hosts on the network are known to be infected with malware, and encrypted DoH traffic is coming from those hosts, it could be a sign that the malware is using DoH to communicate with its command and control servers [16].
- DoH traffic bypassing DNS filters: if the implemented DNS filters block access to known malicious sites and DoH traffic sidesteps these filters, it could be a sign that someone is using DoH to bypass the implemented DNS filters [17].

To detect malicious DoH traffic, the system may need to implement specialized tools and techniques, such as deep packet inspection [18,19], behavioral analysis [20,21], or machine learning algorithms [13,22]. Additionally, it is important to stay up-to-date with the latest threats and vulnerabilities related to DoH and to follow best practices for securing your network and endpoints [23].

User Datagram Protocol (UDP), an unreliable delivery protocol, was the foundation upon which the Domain Name System (DNS) was first created. At the time, the DNS design’s security met all of the requirements of the Internet. However, the strategy is subject to network protocols for current Internet traffic since it offers name-to-address mapping services for the chain of Internet connectivity [24]. Internet-connected networks have developed much more quickly than any other technology globally. The Domain Name System (DNS) has existed on the Internet since its inception and has always been crucial. DNS’ leads users to requested computers, programs, and data by converting domain names to corresponding IPs. Due to the vulnerabilities in DNS system, attackers may use DNS-based malware, DNS amplification, false-positive triggering, DNS tunneling, etc.. To counter these kinds of issues, Google and Cloudflare recently developed and implemented DNS over TLS (DoT) and DNS over HTTPS (DoH) [25]. The DoT and DoH standard protocols encrypt DNS traffic between users and DNS resolver servers to provide privacy and security [26].

Two protocols, DNS over TLS and DNS over HTTPS, were created to encrypt plaintext DNS communication and shield it from the prying eyes of adversaries, ISPs, and other third parties. Keeping with the analogy, these guidelines seek to enclose all postcards sent through the mail, allowing anyone to send a postcard without being concerned that someone is keeping tabs on what they are doing [27,28]. Malicious DNS over HTTPS (DoH) is a type of cyberattack that involves using encrypted DNS over HTTPS to bypass traditional network security measures and send DNS queries to a malicious server controlled by an attacker. Machine learning can improve the effectiveness of malicious DoH attacks by creating more sophisticated and evasive attacks [20].

Using machine learning, attackers can develop more sophisticated methods of evading detection by security tools, such as using randomized domain names, varying the frequency and volume of DNS traffic, and altering the timing of DNS requests. Machine learning can also identify vulnerabilities in specific network configurations and adapt malicious DoH attacks to take advantage of those vulnerabilities [29].

Using machine learning in malicious DoH attacks presents a significant challenge for defenders. It allows attackers to develop more effective and adaptive attack techniques that are more difficult to detect and defend against. However, organizations can protect themselves against malicious DoH attacks by implementing security measures such as monitoring network traffic, implementing threat detection tools that use machine learning, and using encryption and authentication technologies to secure their DNS traffic. Additionally, organizations can invest in training and education programs to ensure their employees know the risks and can identify and report suspicious activity [15,17].

Malicious actors can use DoH to hide their malicious activities from security tools that rely on traditional DNS protocols for detection. Since DoH encrypts DNS queries, it becomes difficult for security tools to analyze DNS traffic for malicious activity. Malicious actors can use DoH to exfiltrate data from a compromised system. Since DoH encrypts DNS queries, it becomes difficult for security tools to detect and block data exfiltration attempts. Malicious actors can use DoH to bypass security controls that rely on DNS-based blacklists and filtering. Since DoH encrypts DNS queries, it becomes difficult for security tools to block access to malicious domains or IP addresses.

Identifying malicious DNS over HTTPS (DoH) traffic can be challenging, but a hybrid learning method can help. The hybrid learning method combines machine learning algorithms with human expertise to detect and classify malicious traffic accurately. A hybrid learning method can accurately detect and classify malicious DoH traffic by combining machine learning algorithms with human expertise. However, it is important to note that this approach requires significant data and expertise to implement effectively.

## 3. Literature Review

Authors in [13] evaluated five standard ML models, including K-nearest neighbors (KNN), C4.5 Decision tree (DT), Random Forest (RF), and Naive Bayes (NB), to work on detecting DoH traffic and analyzing the information obtained from the properties of the protected HTTPS connections. A CIRA-CIC-DoHBrw-2020 dataset, Naive Bayes (NB), and an Adaboost Decision tree were all used. As it could detect DoH traffic using RF classifiers with an accuracy of roughly one (99.99%), the findings of ML approaches were encouraging.

Authors in [17] employed five classes from the CIRA-CIC-DoHBrw-2020 massive cybersecurity data collection from the UNB site as the subject of the study: RNN, RFC, DTC, LSTM, and GRU. They also used GBC, KNC, and XGBoost. Accuracy, MAE, MSE, classification tables, and confusion matrices were additional evaluation metrics.

The results show that for the malicious DoH traffic, XGBC and RFC have achieved the highest accuracy and F1-score. The accuracy rate for the XGBC model was 99.22%. Moreover, RFC had an accuracy of 99.11%. Moreover, the GBC algorithm’s accuracy was 99%. The XGBC, RFC, and GBC models obtained the lowest MAE values of 1.13%, 1.22%, and 1.45%, respectively. Regarding MSE, the GRU model had the lowest error at 1.26%, followed by the RNN model at 1.28%, the LSTM model at 1.28%, and the XGBC model at 1.83%. Also, compared to the findings of the other models, the XGBC model correctly identified 24,793 samples, the largest number. Just behind XGBC, 24,787 samples accurately identified for all four classes came in second place.

The studies show that the XGBC and RFC classifiers perform the best in this data collection. Although the authors of [17] aimed to categorize different DNS tunneling technologies to launch malicious DoH traffic, the data’s lack of diversity poses a con-straint. Also, new methods of data theft are being created quickly. As a result, it is essential to account for the wide range of tunneling assaults. Further research is required to examine the proper procedures to make these tunnels safe and to employ a large-scale data set with as many potential tunnels as possible.

The authors of [22] reduced unnecessary noise, implemented feature selection techniques, and offered explainable features, demonstrating more accurate and effec-tive identification of fraudulent DoH traffic. This work aims to advance previous re-search and create a more suitable model for practical application by eliminating noise, applying feature selection techniques, and emphasizing feature explainability. The results show that the light gradient boosting machine (LGBM) produced the highest accuracy-to-training time ratio, reaching 0% error utilizing 20 top features after removing five overfitting features.

The precise identification of DoH is the focus of [25] discussion of the potential of encrypted traffic analysis. The objective is to assess whether machine learning can extract any information from HTTPS extended I.P. traffic data. To identify the top DoH classifiers, we examined five widely used ML techniques. The results of the studies indicate that DoH recognition is accurate to over 99.9%. Also, as the authors have found (using produced datasets) substantial differences in the behavior of Firefox, Chrome, and Cloudflare, it is possible to identify the application used for DoH communication. With a 99.9% accuracy rate, our trained classifier can identify between DoH clients.

Given the relative youth of the DoH protocol, several earlier works on shielding users against rogue connections have been published. Most earlier research focused on identifying DoH connections since, as they have already mentioned, employing this protocol means that current security mechanisms are bypassed, making it the best security practice to recognize and prevent these connections. Authors in [26] focused on identifying the dangers of utilizing the DoH protocol, outlined the approaches for spotting DoH traffic, and proposed a neural network approach for seeing DoH traffic. They employed a dataset acquired from edge routers through IPFIX/NETFLOW, and the achieved prediction accuracy was 80% for non-normalized data and more than 95% for cleaned and normalized data.

The authors in [30] utilized six machine-learning techniques to propose a system-atic two-layer method for identifying DoH traffic and separating benign from malicious DoH traffic. The effectiveness of the proposed approach was evaluated using accuracy, precision, recall, F-score, confusion matrices, ROC curves, and the significance of the features. The findings demonstrated that the LGBM and XGBoost algorithms outperform the competition in practically all classification parameters, achieving a maximum accuracy of 100% in the layer one and layer two classification tests. Out of four-thousand test datasets, the LGBM algorithm correctly identified one DoH traffic test as a non-DoH test. Out of the 34 features taken from the CIRA-CIC-DoHBrw-2020 dataset, it was discovered that source IP is the most important feature for separating DoH traffic from non-DoH traffic in layer one, followed by destination IP. In contrast, only destination IP is a crucial component for LGBM and gradient boosting algorithms to distinguish between benign and malicious DoH traffic at layer two.

The research methods in [31] have been modified by removing overfitting features and building a useful model from generic features to make them more amenable to practical uses. Removing overfitting features and irrelevant data allows for a quicker, more thorough investigation of DoH detection. The authors recommend the LGBM model due to its exceptional classification accuracy and short training time compared to other machine learning classifiers, such as Random Forest, Decision tree, and XGBoost. This model can distinguish between non-DoH and DoH data and malicious and benign DoH traffic. These encouraging findings open new directions for DoH classification research, including how deep learning outperforms LGBM in terms of speed and accuracy [31].

In [32], the authors used a novel machine-learning architecture to construct an explainable AI solution. In particular, they used the publicly accessible CIRA-CIC-DoHBrw-2020 information to develop a precise method for identifying and categorizing DNS over HTTPS assaults. For the given classification challenge, the proposed model achieved extremely high precision, recall, and F-score of 99.91%, 99.92%, and 99.91%, respectively. However, detailed methods and materials should be provided for reproducablity.

In [33], collections of DoH resolvers are connected to Firefox over various test sessions. The collected traffic is next examined for DoH traffic using temporal characteristics and packet sizes. The proposed model detected DoH requests from other HTTPS traffic using factors related to packet size. Also, a preliminary step is demonstrated that enables external listeners to identify requested websites based on trends in DoH packet sizes. Last but not least, this research offers recommendations for improving DoH by padding the inquiries to increase the privacy benefits offered by DoH. The results of this study demonstrate that DNS privacy still confronts difficulties and that a complete examination of the dangers to DoH privacy is necessary.

In [34], authors explored the classification of DNS over HTTPS communication. The majority of linked works make use of various dataset properties. The trained models cannot be generalized to other network environments since certain incorporated attributes are exclusive to some. A machine-learning model’s generalization is crucial since it will influence how well it performs in different network contexts. To aid in generalizing deep learning models, the authors constructed an adequate data processing pipeline to handle the CIRA-CICDoHBrw-2020 time series dataset, including feature selection and data imbalance handling. In addition, they created generalized deep learning models, such as the LSTM and BiLSTM models, to accurately and quickly categorize DoH traffic. Although both models perform well, the BiLSTM model outperforms the LSTM model in accuracy and computation time.

To identify malicious DoH tunneling and create a fully functional DoH detection system that can be integrated with the security operating system of a corporate network, authors of [35] presented a detection system for DoH tunneling attacks based on a transformer model. The suggested system is a simple and effective DoH tunneling detection solution for a business network to successfully detect malicious DoH traffic mixed in with HTTPS traffic. The suggested system offers a distributed collection mechanism that the security operation center (SOC) can use to gather HTTPS data from any network device and analyze it in real-time to look for malicious DoH traffic. Compared to current suggestions, the detecting module with the Transformer model has numerous advantages. A substantially lower amount of labeled data is needed. Even though it is trained using only 25% of the labeled data, it outperforms earlier techniques in terms of accuracy with the same amount of labeled data, up to 99%. To sum up, Table 1 provides a briefing summary for the examined research articles.

## 4. DoH Identification Architecture

**Figure 3:**
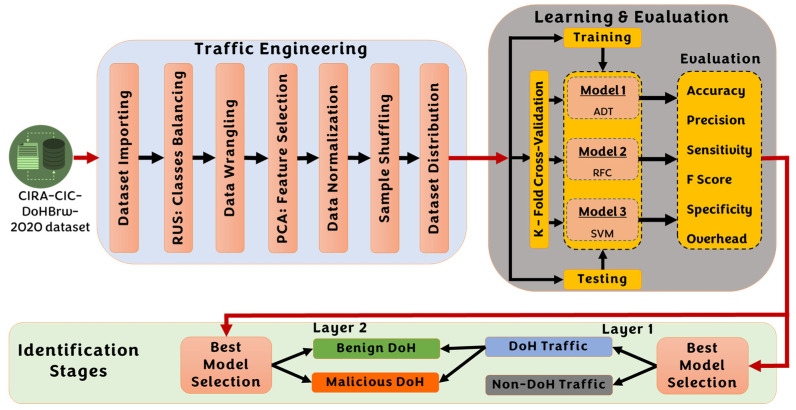
The overall architecture for the proposed double-stage DoH identification system.

DNS over HTTPS (DoH) protocol was originally invented to ensure that attackers are not allowed to falsify or modify DNS traffic. To do so, DoH encrypts all DNS requests/responses before dispatch via HTTPs protocol. It also uses the same standard HTTPS port number (i.e., port number 443) to encapsulate the DNS request in the HTTPS traffic request [36]. Despite all such security mechanisms implemented into the DoH protocol, attackers can still use advanced attack approaches to steal information on the fly through the transmission of malicious DoH traffic. Therefore, like any Attack-Aware defense system [37], we are concerned about detecting malicious DoH Traffic using intelligent supervised methods in this research. Figure 3 illustrates the overall system architecture for the proposed lightweight double-stage scheme to identify malicious DNS over HTTPS traffic using a hybrid learning approach. According to the figure, the proposed system comprises three subsystems: traffic engineering, learning and evaluation, and identification subsystems.

### 4.1. Traffic Engineering Subsystem

**Figure 4:**
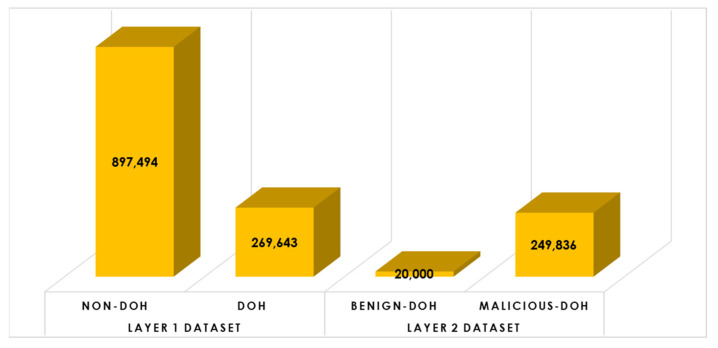
The overall CIRA-CIC-DoHBrw-2020 dataset distribution.

This subsystem involves data preparation and preprocessing activities to set up the data for the learning process. In this research, the CIRA-CIC-DoHBrw-2020 dataset [38] has been employed to evaluate the proposed model for identifying the malicious DNS over HTTPS Traffic (DoH) using supervised learning models. CIRA-CIC-DoHBrw-2020 dataset was originally composed of two datasets: (1) layer one dataset that is used to classify the DNS traffic into either DoH or non-DoH and composed of 269,643 samples for DoH traffic and 897,494 samples for non-DoH traffic. (2) layer two dataset that is used to classify the DoH traffic into either benign-DoH or malicious-DoH and composed of 20,000 samples for benign-DoH traffic and 249,836 samples for malicious-DoH traffic. Figure 4 below illustrates the histogram distribution for the CIRA-CIC-DoHBrw-2020 dataset.

**Figure 5:**
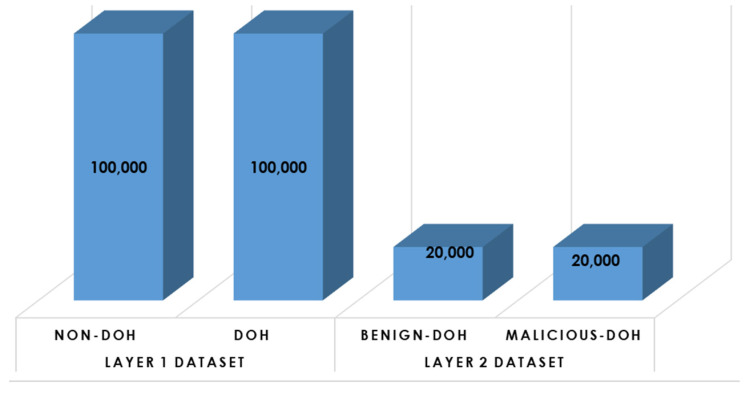
The overall dataset distribution after balancing and under-sampling.

**Figure 6:**
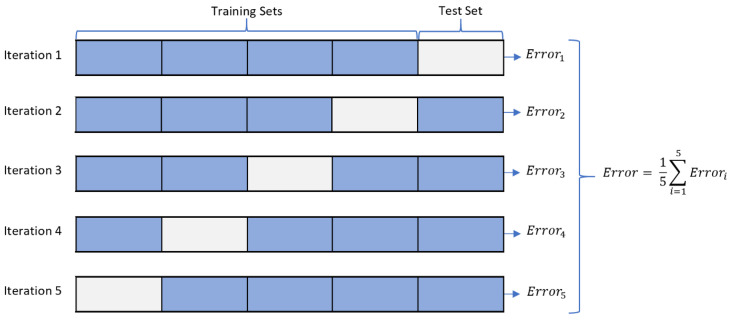
Demonstrating the five-fold cross-validation.

Besides, samples were generated in both datasets using 34 features and one class label. The features and their description can be received from [39]. Once the dataset is collected and imported via MATLAB tables, it will then go through the following consecutive processing stages:

- Class Balancing: Since the CIRA-CIC-DoHBrw-2020 dataset is class-imbalanced, we have employed the random under-sampling (RUS) approach [40] in an attempt to balance all classes in the dataset and to minimize the number of samples in each class. As a result, the dataset for layer one after RUS is composed of 10,000 samples for DoH traffic and 10,000 samples for non-DoH traffic, and the dataset for layer two after RUS is composed of 20,000 samples for benign-DoH traffic and 20,000 samples for malicious-DoH traffic. Figure 5 illustrates the histogram distribution for the balanced-reduced dataset.
- Data Wrangling: this is the process of assuring that data is error-free and ready for use by other learning modules. At data wrangling [41], several activities are applied, including data cleaning from any noisy or mistakenly entered records, eliminating duplications in the samples, filling missing data with (zero, min, max, or mean values), and data exploration to validate the distribution and frequency (using a histogram) for each target label.
- Feature Selection: This procedure reduces the number of input attributes to be supplied and handled by a supervised detection/classification model [42]. This improves the model performance by boosting the prediction speed and minimizing the prediction overhead. In this research, we have employed principal component analysis (PCA) at the preprocessing stages to reduce the dimensionality (reduce the number of input feature sets). PCA decreases the number of dimensions (features) while increasing the interpretation of data and preserving the maximum amount of information [43]. As a result, to satisfy the lightweight performance of our proposed model, we have used the least number of features that maximize the system performance. The final set includes only six out of thirty-four features (FlowBytesSent, FlowReceivedRate, PacketLengthStandardDeviation, PacketLengthMean, PacketLengthMedian, PacketLengthMode).
- Data Normalization: While processing the dataset, we may find some features with values scattered over wide scales. This can negatively affect the classifier’s performance/stability during the training process. Data normalization is employed to overcome this issue by converting features to an analogous scale. The most common way to perform the normalization is the use of the min-max normalization technique [44], which we employ in our research. The normalized value of x ($x_{scaled}$) is given as follows:
  $$X_{scaled}=\frac{X-X_{min}}{X_{max}-X_{min}}$$
- Samples Shuffling: This process involves mixing the data sample (rows) while retaining logical associations between features (columns). This means randomly changing the locations for several samples but keeping the feature values in the same order. Shuffling is essential to eliminate any sort order in the dataset, ensuring the classifier is not overfitting to particular class duo sort order [45].
- Dataset Distribution: This process involves dividing the dataset into training and testing (validation) datasets. In this research, we have used 75% of the dataset for training, while 25% is left for testing the model using five-fold cross-validation to evaluate the model’s performance for all folds of data in the dataset. Five-fold cross-validation is commonly used to eliminate classifier biasing toward one of the target classes during the validation process [46]. Figure 6 illustrates the process of five-fold cross-validation. The performance metrics are calculated as the average of the five experiments’ results (five folds) [47].

In fact, we have conducted extensive validation experiments using different distribution ratios, including 90:10, 80:20, 70:30, 60:40, 50:50, and even 60:40. We have obtained very similar results in almost all experiments to these reported in our article. Therefore, to ensure highly validated, unbiased, and comprehensive/rigorous validation, we have instead performed and reported the results of five-fold cross-validation. The cross-validation process involves training the machine learning model K times (i.e., five times in our case), each using a different fold as the validation set and the remaining folds as the training set. This means that each data point in the original dataset is used for both training and validation at least once. Overall, K-fold cross-validation is an important technique for evaluating machine learning models’ performance and can help ensure that models are generalizable and not overfit to the training data.

### 4.2. Learning and Evaluation Subsystem

**Figure 7:**
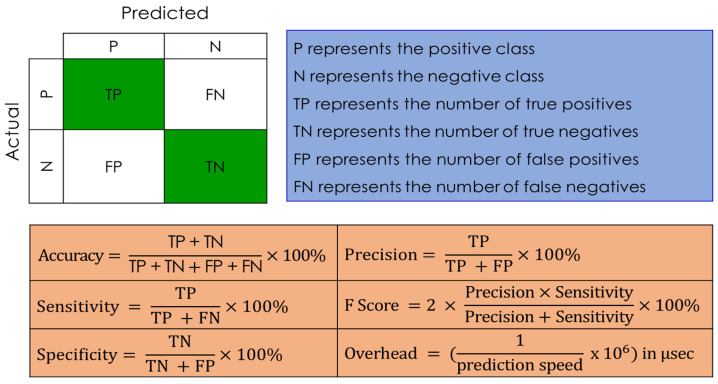
Summary of performance evaluation metrics.

Once the data is preprocessed and prepared, it can be supplied to the next learning and evaluation subsystem. According to the system architecture shown in Figure 3, we have developed and evaluated our system using three main powerful supervised learning approaches: the Adaboost trees (AD) [48], random fine trees (RFC) [49], and the support vector machine (SVM) [50]. To evaluate the performance of each model, we have used the standard machine learning evaluation metrics summarized below in Figure 7.

### 4.3. Traffic Identification Subsystem

Once the learning models are developed, trained, tested, and evaluated, the best model among the three models is selected to perform the identification operation at every layer. The first layer examines the traffic to identify either DoH or non-DoH traffic. If the traffic belongs to DoH traffic, then, at the second layer, the DoH traffic is further investigated to be identified as benign DoH or malicious DoH. The implemented learning models have been evaluated for each layer independently. As we will see in the next section, each layer will finally be deployed with only one computational intelligence model to provide autonomous functionality for each traffic sample.

## 5. Results and Discussion

**Figure 8:**
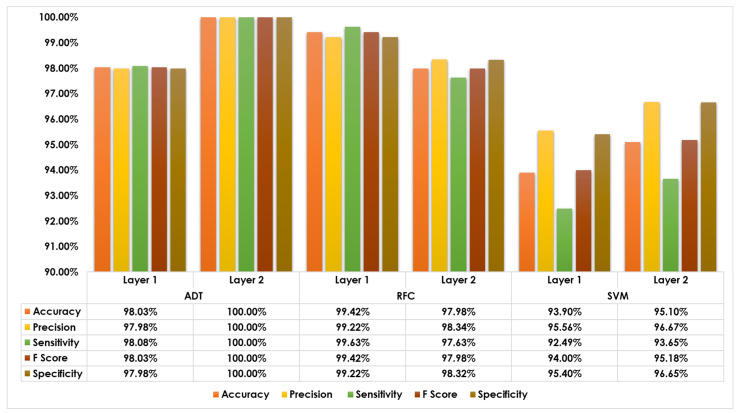
Performance evaluation of several learning models.

System evaluation is a crucial stage in any system development life cycle. It provides key insights and indicators about the system’s capabilities and measures the final system (or the different design alternatives) against its initial performance goals. In this paper, the main goal of our research is to develop an intelligent, autonomous, and lightweight intrusion detection system to Identify malicious DoH Traffic with competent performance factors. Therefore, we have developed different development alternatives, and here in this section, we evaluate all of them in order to pick up the model that satisfies our design goals. To sum up, Figure 8 demonstrates a comparative illustration with tabulated outcomes for the three design alternatives (ADT, RFC, and SVM) implemented for every layer (layer one and layer two) in terms of five performance indicators (accuracy, precision, sensitivity, specificity, and F-score). For layer one, the bar charts show the superiority of RFC based model scoring the greatest performance indicators. For layer two, the bar charts show the superiority of ADT based model scoring the greatest performance indicators. Therefore, the RFC and ADT models have been selected to implement layer and layer two, respectively, for the target DoH identification system. In addition, it is also worth mentioning that we have calculated the prediction overhead for every model, and all models exhibited lightweight predictability with only 2.27 µs, 0.83 µs, and 1.96 µs of inferencing time for ADT, RFC, and SVM, respectively. Eventually, the rest of the results will concentrate on the selected models at each layer to gain more insights into the solution approach and the final deployed system operation and capability.

**Figure 9:**
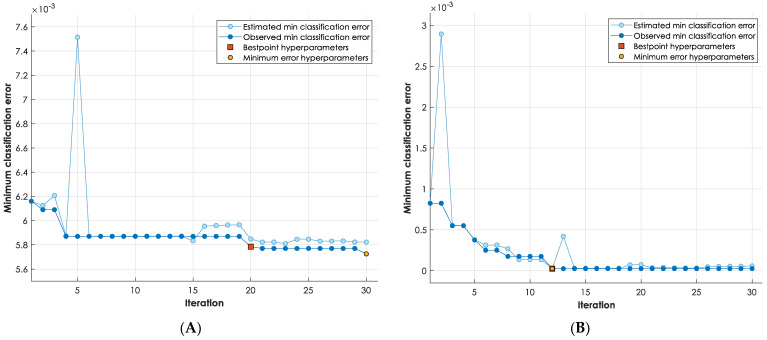
Minimum classification error (%) vs. iteration number (1–30): (**A**) layer one performance trajectory using an RFC model, (**B**) layer two performance trajectory using an ADT model.

According to the aforementioned evaluation and analysis, the traffic is examined using random fine trees (RF) to identify either DoH or non-DoH traffic at the first layer. At the second layer, the DoH traffic is further investigated using Adaboost trees (AD) to be identified as benign DoH or malicious DoH. In Figure 9, we trace the performance trajectories for the model implemented at layer one and layer two in terms of minimum classification error (MCE) vs. learning iteration (1–30 iterations). The learning curves display a stable tendency in both cases while minimizing the minimum classification error at every learning iteration. The best point/minimum error hyperparameters have been recorded and stabilized at iteration twenty for the layer one model and iteration twelve for the layer two models scoring an MCE of 0.6% and 0.0% for the layer one model and layer two models, respectively. This complies with the accuracy values obtained earlier (accuracy = 1 − MCE%) for every model (99.6% for the RFC model at layer one and 100% for the ADT model at layer two).

**Figure 10:**
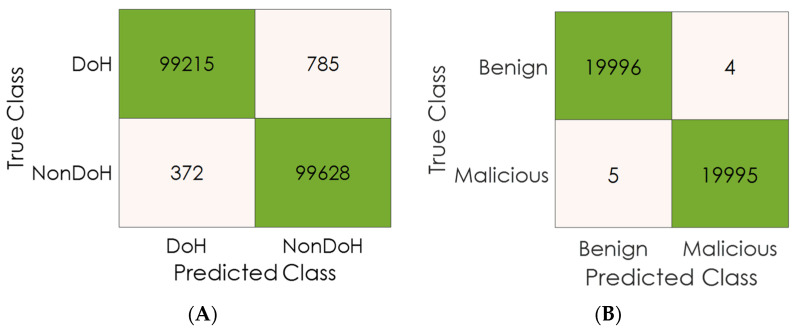
Confusion matrix analysis: (**A**) Layer one results, (**B**) Layer two results.

Also, Figure 10 shows the confusion matrix analysis for layer one (RFC-based model) and layer two (ADT-based model). We can obviously infer that the classification cost for layer one is 1157 misclassified samples (FP + FN) out of 200,000 (total number of samples), while the classification gain for layer one is 198,843 correctly classified samples (TP + TN) out of 200,000 (total number of samples). On the other hand, we can infer that the classification cost for layer two is nine misclassified samples (FP + FN) out of forty-thousand (total number of samples). In comparison, the classification gain for layer one is 39,991 correctly classified samples (TP + TN) out of 40,000 (total number of samples).

**Figure 11:**
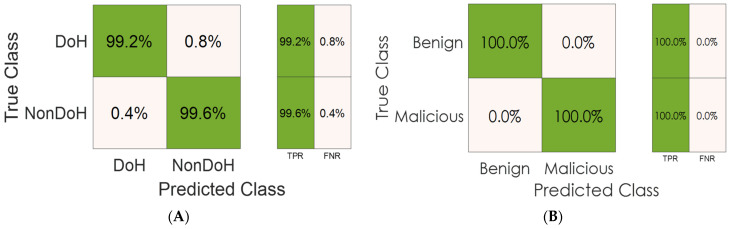
Sensitivity matrix analysis: (**A**) layer one results, (**B**) layer two results.

Moreover, Figure 11 shows the sensitivity matrix analysis for layer one (RFC-based model) and layer two (ADT-based model). This matrix analyzes the true positive rate (TPR) and the false negative rate (FNR) for each class in the datasets corresponding to each layer. We can obviously infer that all classes show high sensitivity toward identifying the traffic classes scoring high TPRs of 100% or nearby.

**Figure 12:**
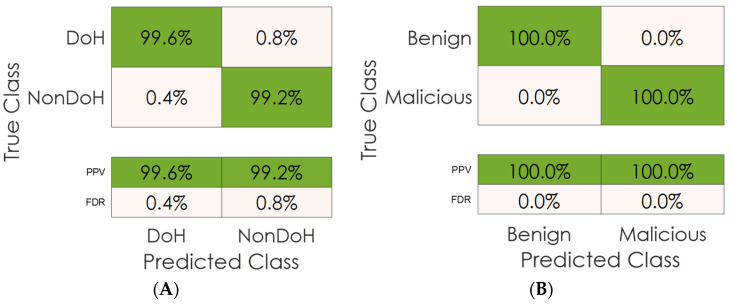
Precision matrix analysis: (**A**) layer one results, (**B**) layer two results.

Furthermore, Figure 12 shows the precision matrix analysis for layer one (RFC-based model) and layer two (ADT-based model). This matrix analyzes the positive predictive value/rate (PPV) and the false discovery rate (FDR) for each class in the datasets corresponding to each layer. We can apparently imply that all classes show high precision and discovery rates toward identifying the traffic classes scoring high PPVs of 100% or nearby.

Finally, Table 2 contrasts our best-performant system results with similar state-of-the-art systems employing the same/similar dataset. The assessment in this table considers several compassion factors, including the employed learning technique, the number of utilized features (input features set), the number of samples (instances) used to train and validate the learning models, the predictive overhead (time), the detection/identification accuracy (%) and the detection/identification F Score (%). It can be clearly seen that our proposed model is very notable with a lightweight and high detectability process. It is also worth mentioning that even though many existing models have achieved high identification accuracy rates, however, our system has a great advantage over these models by achieving similar or even higher accuracy rates with only six features out of thirty-four (only ~18% of the features were used) and a smaller number of samples. Only 240,00 samples out of 1,436,973 (only ~17% of the features) were used. This, in turn, came up with a much less complex system that can operate at a very smaller prediction overhead, and thus it is a lightweight system. According to the comparison stated in Table 2, diverse learning models have been presented in the literature to address the problem of identifying malicious DoH. These include (but are not limited to) the long short-term memory (LSTM) based DoH identification model proposed by Davidson et al. [38], the one class support vector machine (OCSVM) based DoH identification model proposed by Mbona et al. [51], the Bidirectional-LSTM (Bi-LSTM) based DoH identification model proposed by X. Du et al. [52], the ensemble learning of Decision trees (EL-DT) based DoH identification model proposed by Chijioke et al. [53], the optimizable K-nearest neighbors (O-KNN) based model proposed by Al-Haija et at. [54], the LSTM-based DoH identification model proposed by C. Yue et al. [55], the Xgboost Classifier (XGBC) based DoH identification model proposed by Rafa et al. [56], the Random Forest classifier (RFC) based DoH identification model proposed by Y. Li et al. [57], the semi-supervised support vector machine (SSSVM) based DoH identification model proposed by Nguyen et al. [58], and finally, the Fuzzy clustering (Fuz-CL) based DoH identification model proposed by Dang et al. [59].

## 6. Conclusions and Future Work

Although it has security features, such as an IP blacklist and a DNS firewall, the Domain Name System (DNS) still has privacy problems because it is a plaintext communication. An encrypted DNS known as DNS-over-HTTPS (DoH) has recently been created to address this issue and is becoming increasingly popular. Supervised machine learning techniques were previously employed in research to identify DoH tunneling, which needed a large amount of labeled data. In reality, gathering and categorizing every piece of data is impractical, especially in the DoH, where every piece of data is encrypted. This research offers a novel hybrid learning-based double-stage strategy to detect malicious DoH traffic. The system is divided into two layers. The first layer employs random fine trees (RF) to determine whether it is DoH traffic. The DoH traffic is further examined at the second layer using Adaboost trees (AD) to distinguish between benign and malicious DoH. The proposed system is lightweight because it uses the fewest features (6 out of 33 characteristics), chosen using principal component analysis (PCA), and the fewest samples possible, which are created using a random under-sampling (RUS) method. The experiential evaluation identified a high-performing system with layer one and layer two identification prediction overheads of 0.83 µs and 2.27 µs, respectively, and predicted accuracy of 99.4% and 100%. Our near-future plans are focused on developing unsupervised machine learning-based DoH IDS to alleviate the limitation of the labeled data requirement. Also, efficient feature augmentation and engineering techniques [60] can be applied at the preprocessing stages to improve the data preparation process prior to the learning phases.

## Figures and Tables

**Table 1:** Briefing table for the examined research articles.

Ref.	Method	Limitations
[30]	LGBM and XGBoost algorithms	Requires a huge number of labeled datasets
[22]	LGBM	Overfitting and time-consuming to filter data and eliminate the noise
[32]	Balanced and stacked Random Forest	The model is inefficient and too slow to make real-time predictions.
[17]	RNN, RFC, DTC, LSTM, GRU, They used GBC, KNC, and XGBoost	Require a huge amount of labeled data
[33]	Multi-Layer Perceptron	The model’s structure is complex, and real-time performance is poor.
[34]	LSTM model	Model complexity and more training data required to learn effectively
[13]	K-nearest neighbors (KNN), C4.5 Decision tree (DT), Random Forest (RF), and Naive Bayes (NB)	Time-consuming, requires a huge number of labeled datasets, and the real-time performance could be better.
[25]	K-nearest neighbors, C4.5 Decision tree, Random Forest, Naïve Bayes, and Adaboost Decision tree	Due to similarities with other requests and responses, the suggested ML algorithm cannot identify a DoH connection with a single query.
[35]	Transformer	Requires a high volume of labeled data, more complex than other models

**Table 2:** Comparing our results with existing systems.

Ref.	Model	# of Features	# of Samples	Overhead	Accuracy	F1 Score
Davidson et al. [38]	LSTM	34	1,436,973	20.4 µs	99.0%	98%
Mbona et al. [51]	OCSVM	34	1,436,973	-	-	85%
X. Du et al. [52]	Bi-LSTM	34	1,436,973	1.14 ms	99.6%	99.6%
Chijioke et al. [53]	EL-ADT	34	1,436,973	-	99.5%	-
Al-Haija et al. [54]	O-KNN	34	141,530	100 µs	97.1%	97.3%
C. Yue et al. [55]	LSTM	34	1,436,973	>1 ms	97.5%	-
Rafa et al. [56]	XGBC	34	1,436,973	-	99.2%	
Y. Li et al. [57]	RFC	34	1,436,973	-	98.5%	98.5%
Nguyen et al. [58]	SSSVM	34	1,436,973	-	94.0%	93.2%
Dang et al. [59]	Fuz-Cl	34	1,436,973	-	85.0%	87.0%
**This work**	**Hybrid**	**6**	**240,000**	**3.5 µs**	**100%**	**100%**

## Data Availability

Data associated with this research can be retrieved online from the Canadian Institute for Cybersecurity via https://www.unb.ca/cic/datasets/dohbrw-2020.html (accessed on 19 December 2022).

## Abbreviations

The following table lists the full forms of abbreviations used in this paper.

ADT	Adaboost trees
BiLSTM	Bidirectional LSTM
DNS	Domain Name System
DoH	DNS over HTTPS
DoT	DNS over TLS
DT	Decision trees
DTC	Decision trees classifier
EL-ADT	Ensemble learning of Adaboost trees
EL-DT	Ensemble learning of Decision trees
FDR	False discovery rate
FN	False negative
FNR	False negative rate
FP	False positive
Fuz-CL	Fuzzy clustering
GBC	Gradient boosting classifier
GRU	Gated recurrent units
HTTPS	Hypertext transfer protocol secure
IDC	International data corporation
IDS	Intrusion detection system
IP	Internet protocol
IPFIX	Internet protocol flow information export
ISPs	Internet service provider
KNC	K-nearest classifier
KNN	K-nearest neighbors
LGBM	Light gradient boosting machine
LSTM	Long short-term memory networks
MAE	Mean absolute error
MCE	Minimum classification error
MSE	Mean squared error
NB	Naive Bayes
NETFLOW	Network traffic flow
O-KNN	Optimizable K-nearest neighbors
OCSVM	One class support vector machine
PCA	Principal component analysis
PPV	Positive predictive value
RFC	Random Forest classifier
RNN	Recurrent neural networks
RUS	Random under-sampling
SOC	Security operation center
SSSVM	Semi-supervised support vector machine
TLD	Top-level domain
TLS	Transport Layer Security
TN	True negative
TP	True Positive
TPR	True positive rate
UDP	User Datagram Protocol
UNB	University of New Brunswick
XGBC	eXtreme Gradient Boosting
